# Using egocentric analysis to investigate professional networks and productivity of graduate students and faculty in life sciences in Japan, Singapore, and Taiwan

**DOI:** 10.1371/journal.pone.0186608

**Published:** 2017-10-18

**Authors:** Noriko Hara, Hui Chen, Marcus Antonius Ynalvez

**Affiliations:** 1 Department of Information and Library Science, School of Informatics, Computing & Engineering, Indiana University, Bloomington, Indiana, United States of America; 2 Department of Sociology, Indiana University, Bloomington, Indiana, United States of America; 3 Department of Social Sciences, Texas A&M International University, Laredo, Texas, United States of America; Kyoto University, JAPAN

## Abstract

Prior studies showed that scientists’ professional networks contribute to research productivity, but little work has examined what factors predict the formation of professional networks. This study sought to 1) examine what factors predict the formation of international ties between faculty and graduate students and 2) identify how these international ties would affect publication productivity in three East Asian countries. Face-to-face surveys and in-depth semi-structured interviews were conducted with a sample of faculty and doctoral students in life sciences at 10 research institutions in Japan, Singapore, and Taiwan. Our final sample consisted of 290 respondents (84 faculty and 206 doctoral students) and 1,435 network members. We used egocentric social network analysis to examine the structure of international ties and how they relate to research productivity. Our findings suggest that overseas graduate training can be a key factor in graduate students’ development of international ties in these countries. Those with a higher proportion of international ties in their professional networks were likely to have published more papers and written more manuscripts. For faculty, international ties did not affect the number of manuscripts written or of papers published, but did correlate with an increase in publishing in top journals. The networks we examined were identified by asking study participants with whom they discuss their research. Because the relationships may not appear in explicit co-authorship networks, these networks were not officially recorded elsewhere. This study sheds light on the relationships of these invisible support networks to researcher productivity.

## Introduction

Many things influence scientists’ productivity. One contributing factor is a scientist’s professional network [1–4], which has increased in importance as research requires ever more extensive assistance from fellow scientists. This assistance may lead to more explicit collaboration and, in turn, to co-authorships. The steady increase in the number of co-authored papers documents the growing reliance on research teams [5–7]. Co-authored papers tend to be published in higher impact journals [8, 7] and are more likely to be cited [9, 10]. From this, one can infer that co-authored papers tend to be higher quality, arguably because more individuals’ ideas are included, and that the higher citation of co-authored publications may reflect the wider readership by reaching each author’s personal network. In addition, certain kinds of research require access to special equipment and related expertise, thus increasing collaboration, especially in the hard sciences such as the biological and chemical sciences.

Collegial advice, another type of scientific collaboration, may be provided through, for example, comments on manuscripts or suggestions on research direction. It entails less involvement and yet is still important for knowledge production. This type of collaboration is frequently invisible because it does not appear as co-authorship. However, it is sometimes credited in acknowledgments. Cronin [11] examined acknowledgments in five prominent information science journals and reported that the number of articles with acknowledgments consistently increased between 1971 and 1999. He concluded that this trend is plausibly a result of reliance on trusted colleagues for feedback and a “network of collaborators” (p. 432). Because both strong and weak scientific collaboration affect a researcher’s productivity, investigating their professional networks can improve understanding of knowledge production.

Advances in information and communication technologies have facilitated scientific collaboration across multiple countries in recent years [12]. International collaboration, in particular, tends to produce highly cited publications [13, 14] and leads to papers published in respected venues [15].

In this paper, we address less explicit scientific collaboration and examine egocentric social networks of faculty and graduate students in life sciences in Japan, Singapore, and Taiwan. We asked them to name individuals from whom they received advice and with whom they discussed research. The scientists in these three countries have been reported to have high productivity [16, 17]. We sought to identify what factors, if any, influence the publication productivity of these scientists; specifically: 1) what factors influence the formation of international ties (versus domestic ties) in the discussion networks and 2) whether and how international ties influence publication productivity.

### Social network and productivity

The majority of prior studies in this area examined researchers’ professional networks through co-authorship analysis, i.e., strong collaboration. This line of research revealed, for example, that researchers in a bridging position tend to be more productive [18, 19] and that researchers who have more co-authors and one frequent, primary co-author tend to score high in productivity [18]. Using publication records of co-authorship, Petersen [20] found that two thirds of the collaboration ties were weak, lasting less than five years, and that “a remarkable 60–80% of collaboration lasted only one year” (p. E4679). At the same time, Petersen’s study indicated that super ties, or extremely strong ties, were common, including approximately one in 25 researchers. Networks with these super ties were found to be more productive.

Few studies have looked at researcher’s social networks that include weak collaboration. Taba, Heard, Brennan, Lee, and Lewis [21] investigated professional networks of Australian radiologists using egocentric social network analysis. In addition to a survey about their professional work, the radiologists read a test set of mammograms. The study found that strong ties appeared to help radiologists develop better analytical skills, specifically, to interpret medical images accurately. With the combination of bibliometric and egocentric social network data, Wang [22] found that strong ties positively affect researchers’ productivity at first, but eventually have a negative impact when tie strength becomes too strong. In Wang’s study, tie strength was measured by the frequency of collaboration within five years.

### Global scientific collaboration

Scientific collaboration is not limited by geographical boundaries. Scientists all over the world reach out to their international colleagues and the globalization of scientific collaboration has steadily and significantly increased [12, 23]. Waltman et al. [12] studied distances separating collaborators on 21 million research publications from various countries and fields of science and found that the average distance was 1,553 kilometers in 2009, increased from 334 kilometers in 1990. Although collaborations that cross national and geographical boundaries are not easy, such scientific collaboration tends to result in higher citation counts than the publications by strictly domestic collaborators according to Glänzel and Schubert [13], who studied citation impacts of international co-authorship in chemistry. Similarly, Hong and Zhao [15] found that Chinese scientists with network contacts overseas were more likely to publish papers indexed in the *Science Citation Index*. Findings such as these validate government initiatives, such as in Finland, to support globalization in science [14].

Coccia and Wang [24] conducted a longitudinal bibliometric study to examine how international scientific collaboration evolved from 1973 to 2012. They found that international collaboration in physics and mathematics, i.e., basic research fields, had declined, whereas in biology and clinical medicine, i.e., applied research fields, it had increased. They also reported that international collaboration had generally increased over time, although the percentage of international collaboration with institutions had not changed. In other words, the basic structure of collaboration in terms of institutional level versus individual level has not altered.

Guerrero Bote, Olmeda-Gomez, and de Moya-Anegon [25] investigated advantages of international collaboration using bibliographic data in the Scopus database. They found that papers with international collaborators tend to be cited more often than papers with domestic researchers and that, in general, the higher the number of collaborating countries on an article, the more citations it accumulates. Interestingly, the authors noted that the U.S. gains the fewest citations from international collaboration because U.S. institutions already have diverse researchers and rich resources. Asian countries, such as China, India, South Korea, and Japan, tend to produce many publications but overall score lower on the percentage of international collaboration and normalized citations. Guerrero Bote et al. [25] speculated that this may be a consequence of persistent language barriers, even though these countries are producing more scientific publications.

Prior research shows that both international collaboration and the proportion of foreigners in scientists’ discussion networks is positively associated with productivity [26]. Ynalvez and Shrum [26] examined the relationship between professional networks, scientific collaboration, and publication productivity among scientists in research institutions in the Philippines. Graduate training overseas (i.e., in developed countries) is positively associated with the proportion of a scientist’s contacts in those countries. Researchers’ strong international ties in the former host country can serve as a prerequisite or a driver of international research collaboration, which can contribute to publication productivity.

Few studies have examined the formation of social networks themselves. In this paper, we seek to fill this gap by investigating factors associated with the formation of research discussion networks, i.e., weak collaborations, focusing on international ties in the network. We refer to a tie as “international” when scientists discuss research with colleagues living abroad, and as “domestic” when the discussions are with colleagues living in the same country. First, we ask what makes people choose discussion partners who live abroad rather than in the same country. Second, we ask whether and how the international ties in the discussion network influence productivity.

## Methods

### Ethics statement

Both Indiana University IRB (Study # 08–13318) and Texas A&M International University IRB (Study # 2008-06-22) have approved the study. The informed consent form was presented to all participants, and written consent was received when participants agreed to respond to the survey and interview questions.

### Sample

Face-to-face surveys and in-depth semi-structured interviews were conducted among a sample of faculty and doctoral students in the life sciences at 10 research institutions in Japan, Singapore, and Taiwan. In each country, 30 faculty and 70 doctoral students were interviewed. Faculty and students were randomly drawn from list frames generated from departmental websites of the target institution. The survey questionnaire and interview included questions on research productivity, research projects, research involvement, research discussion network, and socio-demographic characteristics. Questions on the research discussion network employed an egocentric social network design in the form of a name generator with its attendant name interpreter (more detailed descriptions are presented in the Measures section). Each respondent (i.e., ego) could nominate up to 10 individuals (i.e., alters) with whom the ego discussed important matters about research. Our final sample included a total number of 290 respondents (84 faculty and 206 doctoral students; 100 respondents came from Japan, 90 from Singapore, and 100 from Taiwan) and 1,435 network members. For each interview session, respondents were interviewed in either their native language or English. Interviews were recorded and lasted about an hour on average.

## Measures

### Name generator instruments for egocentric networks

Surveys have long employed name generator instruments to collect egocentric network data. Instruments typically include two types of questions: name generators that ask the names of people in the respondent’s social network (herein called alters), and name interpreters that ask information on the characteristics of the alters and the relationships between the respondent and alters [27, 28]. In the questionnaire of our study, respondents were asked to nominate up to 10 alters whom they talk to, go to for advice, or who come to them for advice regarding research. For each alter, the following questions were asked: the alter’s gender, nationality, location, types of relationship between the alter and respondents (e.g., graduate advisor, colleague, graduate student, and spouse), duration of the relationship, and communication mode (e.g., face-to-face, cell phone, and email).

### International ties

International ties are measured based on information from a name generator and name interpreter of ego’s network mentioned above. Respondents were asked to nominate up to 10 alters with whom they discuss research. For each alter, there is a question on the alter’s location, and the responses had 10 categories ranging from “in the department” and “in another institution within the country” to “other countries.” We collapsed the responses into two categories: within the country and outside of the country. According to this categorization, we created a dummy variable. Ties with alters living outside of the country are referred to as international ties and coded as 1, and ties with alters living within the country are referred to as domestic ties and coded as 0. This variable was used as the outcome in the multilevel logistic regression models to answer the question about what characteristics of alters, ties, egos, and networks influence the formation of international versus domestic ties.

### Research productivity

In the questionnaire, respondents were asked how many manuscripts (published or unpublished) they have written in the last 12 months, and how many articles they have published in international, national, and top journals respectively during the last 3 years. Based on these questions, we constructed three variables to measure research productivity: 1) the number of manuscripts written in the last 12 months, 2) the number of papers published in top journals, and 3) the total number of papers published (i.e., papers published in both international and national journals). The last two variables were based on the time period from 2007 to the time when the surveys were conducted (in 2010).

Top journals are defined as journals with an impact factor of at least four (≥ 4). These three measures of research productivity were used as the outcomes in the regressions to answer the question of whether and how international ties influence research productivity.

### Alters and ties

Characteristics of alters and ties include the alter’s gender (1 = female, 0 = male), the ego’s relationship with the alter (1 = mentor-mentee, 2 = peer, 3 = partner, 4 = other), the intensity of the discussion (measured by the time spent in discussion), how long the ego has known the alter (in years), and the mode of communication (1 = conversational only, e.g., face-to-face, phone, and Skype, 2 = non-conversational only, e.g., email, instant messaging, and chat room, 3 = both). The mode of communication was inspired by Turkle [29], who encouraged attention on different types of communication and whether they require participants to engage in spontaneous interactions (i.e., conversation) rather than mostly relying on asynchronous communication.

### Egos and networks

Characteristics of egos include their socio-demographic attributes, such as gender (1 = female, 0 = male), age (in years), and marital status (1 = married, 0 = not married). For professional attributes, we included the percentage of time devoted to research, the number of international conferences attended, and a dummy variable about graduate training abroad (1 = yes, 0 = no) to assess whether the respondent has ever spent time outside of their home country for graduate training.

Measures of network characteristics include network size (total number of alters nominated by the ego), the percentage of women in the network, the percentage of nonconversational-only modes of communication, the percentage of mentor-mentee relationships, the average intensity of discussion in the network (the mean of intensity of discussion aggregated across the whole network), and the average length of time for which the ego has known the alter. These characteristics are aggregated to characterize one’s social networks, which is a standard method in egocentric networks research that uses multilevel models [30].

### Analytical strategy

We use multilevel logistic regression models to answer the first question: what characteristics of egos, alters, ties, and networks are associated with international ties? Because each respondent, or ego, reported several relations with alters, the ego-alter ties are nested within respondents; that is, the structure of the network data is nested or clustered. Traditional statistical methods, such as regression, are not appropriate for data with a hierarchical nesting structure because they assume independence between the ties. With ties nested within egos, they may not be treated as independent. Multilevel models are suitable for data with a nested structure [31]. The level of the ego-alter tie is referred to as level 1, or the lowest level, and the level of the ego is referred to as level 2, or the higher level. Attributes associated with ties and alters are treated as level-1 variables and attributes associated with egos are treated as level-2 variables. The dependent variable in the multilevel logistic regression model is defined at the lowest level, or the level of the ego-alter tie.

This study employs a two-level model to predict the odds that a tie between ego and alter is international rather than domestic. A three-level model in which institutions were included as the third level was examined, but the result showed no improvement in the model’s goodness of fit. Thus, we used only a two-level model in our analysis. The level-1 model (see Eq 1.1) predicts the odds that a tie between ego, i, and alter, j, is international. In this model, β_0i_ is the intercept, β_1i_ the slope, X_ij_ the values of the level-1 variables, i.e., characteristics of alters and ties, and ε_ij_ the error term, which is assumed to follow a normal distribution with a mean of 0 and a standard deviation of σ (see Eq 1.2). The level-1 model aims to examine whether and how an international tie between ego and alter is associated with the attributes of alters and ties, such as an alter’s gender and relationship between ego and alter.

$$\mathrm{Level}\;1\;\mathrm{model}:\;log\;\left(\frac{p_{ij}}{1-p_{ij}}\right)=\beta_{0i}+\beta_{1i}X_{ij}+\varepsilon_{ij}$$ (1.1)

$$\varepsilon_{ij}∼N\left(0,\sigma^{2}\right)$$ (1.2)

Our level-2 model (see Eqs 2.1 and 2.2) is a random-intercept model, that is, only the intercept is allowed to vary randomly over the egos. It shows that the intercept (β_0i_) in the level-1 model depends on level-2 variables, i.e., ego and network characteristics, such as an ego’s gender and the proportion of males in an ego’s network. In this model, Ego_i_ represents the values of the ego characteristics, Network_i_ the values of the egocentric network characteristics, ϒ_00_ the population mean of the intercept (i.e., the fixed effect), and μ_0i_ the random effect, which is assumed to follow a normal distribution with a mean of 0 and a standard deviation of σ_μ_. The slope (β_1i_) includes only the fixed effect (ϒ_1i_).

$$\mathrm{Level}\;2\;\mathrm{model}:\;\beta_{0i}=\gamma_{00}+\gamma_{01}Ego_{i}+\alpha_{01}Network_{i}+{µ}_{0i}$$ (2.1)

$$\beta_{1i}=\gamma_{10}.$$ (2.2)

$${µ}_{0i}∼N\left(0,\sigma_{u}^{2}\right)$$ (2.3)

This level-2 model, when combined with the level-1 model, can be used to examine how an international tie between ego and alter is associated with the attributes of ego and networks, as shown in Eq 3. What is more, all continuous predictors are centered on their grand mean.

$$log\;\left(\frac{p_{ae}}{1-p_{ae}}\right)=\gamma_{00}+\gamma_{01}Ego_{i}+\alpha_{01}Network_{i}+\gamma_{10}X_{ij}+{µ}_{0i}+\varepsilon_{ij}$$ (3)

Because the outcomes are count variables, we use Poisson and Negative Binomial regressions to answer the second question: How do international ties influence research productivity? Poisson regression is usually used for modeling count data. But when the count data exhibits over-dispersion, a situation in which the conditional variance is greater than the conditional mean, negative binomial regression is more appropriate.

## Results

We conducted analyses for faculty and students separately because of the heterogeneity of the two groups.

### Descriptive statistics

Table 1 shows the descriptive statistics of all the variables in our study. With regard to the characteristics of egos, 63% of students and 74% of faculty are male; 19% of students and 77% of faculty are married, and 19% of students and 80% of faculty have been abroad for graduate training. Students range in age from 22 to 43 with the mean being 27.85 (SD = 3.22), whereas faculty range in age from 29 to 68 with the mean being 48.42 (SD = 9.77). On average, students spend 83.61% of their time on research (SD = 14.43) and have written 1.17 manuscripts (SD = 1.30) in the last 12 months, and the figures for faculty are 59.38% (SD = 17.82) and 6.14 (SD = 5.24). The hours that students spent on research is relatively long. This is due to the fact that the graduate curriculum in science in Japan was modeled after the European apprenticeship style in which students rarely take classes, but work on research projects alongside their advisors [32]. In Singapore, although patterned after the U.S. in that it comprises a course work component and a thesis research component, the course work component requires the student to complete only 6 modules (or courses) [personal communication]. As such, the Singaporean graduate students in science spend more time on research than the U.S. students. Compared to these two countries, Taiwan’s graduate curriculum in science is heavily patterned after U.S. institutions given that most faculty and professors in Taiwan have obtained their doctoral degrees in the U.S. [33].

**Table 1:** Descriptive statistics of variables.

Variables	Mean/Prop		SD		Min		Max		N	
	Students	Faculty	Students	Faculty	Students	Faculty	Students	Faculty	Students	Faculty
*Ego*										
Gender (1 = male)	0.63	0.74	-	-	0	0	1	1	206	84
Age (yrs)	27.85	48.42	3.22	9.77	22	29	43	68	206	84
Married (1 = yes)	0.19	0.77	-	-	0	0	1	1	206	84
No. of international conferences attended	0.99	5.17	1.47	5.98	0	0	10	35	206	84
No. of conferences attended	2.48	11.10	2.90	15.29	0	0	13	120	206	84
Graduate training abroad (1 = yes)	0.19	0.80	-	-	0	0	1	1	206	84
Time devoted to research (%)	83.61	59.38	14.43	17.82	30	10	100	100	206	84
Research productivity										
No. of papers published	0.77	11.71	1.29	13.97	0	0	8	90	206	84
No. of papers published in top journals	0.21	3.92	0.59	5.02	0	0	5	29	206	84
No. of manuscripts	1.17	6.14	1.30	5.24	0	0	9	30	206	84
*Alter*										
Gender (1 = male)	0.70	0.77			0	0	1	1	1011	424
Length of time known (yrs)	3.40	9.23	2.97	6.97	0.08	0.25	28	46	991	423
*Ties*										
Cross-national (1 = yes)	0.06	0.17	-	-	0	0	1	1	1011	424
Mode of communication									1010	424
Conversational-only	0.34	0.12	-	-	0	0	1	1	-	-
Non-conversational-only	0.03	0.08	-	-	0	0	1	1	-	-
Both	0.63	0.80	-	-	0	0	1	1	-	-
Relationship									1010	423
Mentor-mentee	0.33	0.22	-	-	0	0	1	1	-	-
Peer	0.45	0.55	-	-	0	0	1	1	-	-
Partner	0.04	0.03	-	-	0	0	1	1	-	-
Others	0.18	0.20	-	-	0	0	1	1	-	-
Intensity of Discussion (mins)	181.42	126.59	252.64	216.83	0	0	1260	1260	1010	417
*Whole networks*										
Network size	4.90	5.03	2.32	2.39	1	1	10	10	206	84
Prop. of men	0.70	0.79	0.26	0.24	0	0	1	1	206	84
Prop. of cross-national ties	0.05	0.19	0.12	0.27	0	0	0.75	1	206	84
Prop. of nonconversational- only mode	0.03	0.09	0.10	0.20	0	0	1	1	206	84
Prop. of mentor-mentee relation	0.37	0.18	0.26	0.29	0	0	1	1	206	84
Mean length of time known (yrs)	3.61	9.53	2.11	5.01	0.17	2.37	14.67	30	204	84
Mean intensity of discussion (mins)	188.32	129.82	179.81	188.10	0	5	840	1260	206	82
Country									206	84
Japan	0.33	0.37	-	-		0		1	69	31
Singapore	0.33	0.26	-	-		0		1	68	22
Taiwan	0.33	0.37	-	-		0		1	69	31

In addition, students have, on average, attended 2.48 conference (SD = 2.90), with 0.99 being international conferences (SD = 1.47), and published 0.77 papers in total (SD = 1.30), with 0.21 papers in top journals (SD = 0.59) from 2007 to 2010. The figures for faculty are 11.10 conferences (SD = 15.29), 5.17 international conferences (SD = 5.98), 11.71 papers in total (SD = 13.97), and 3.92 papers published in top journals (SD = 5.02).

In terms of the characteristics of alters and ties, 70% of students’ alters and 77% of faculty’s are male. On average, students have known each alter for 3.40 years (SD = 2.97) and spend 181.42 minutes (SD = 252.64) in discussion with each alter. For faculty, the figures are 9.23 years (SD = 6.97) and 126.59 minutes (SD = 216.83). Some 6% of students’ ties and 17% of faculty’s are international. For students, 34% of communications are via conversation alone, 3% via non-conversation modes, and 63% involve both conversation and non-conversation. For faculty, the figures are 12%, 8%, and 80%, respectively. Finally, for students, 33% of egos and alters are a mentor-mentee relationship, 45% peers, 4% partners, and 18% others. For faculty, the figures are 22%, 55%, 3%, and 20%.

As for the characteristics of whole networks, network size averages 4.9 for students (SD = 2.32) and 5.0 for faculty (SD = 2.39). Males account for 70% of students’ total networks and 79% of faculty’s. International ties represent 5% of students’ total network ties and 19% for faculty. Only 3% of communications in students’ total networks and 9% in those of faculty’s are limited to non-conversational channels. Mentor-mentee relationships account for 37% of students’ ties and 18% of faculty’s. On average, students have known alters in their total networks for 3.61 years (SD = 2.11) and spend 188.32 minutes (SD = 179.81) on discussion with these alters. The figures for faculty are 9.63 years (SD = 5.01) and 129.82 minutes (SD = 188.10).

### Formation of international ties

Next, we used multilevel logistic models to examine how international ties are associated with characteristics of egos, alters, ties, and networks. Model 1 in Table 2 considers only the effects of ego characteristics on the formation of international ties, controlling for country. For students, marital status and experience in graduate training abroad are significantly associated with international ties. Students who are married are less likely to form international ties than students who are not married (OR = 0.21, p < 0.05). Students who have been abroad for graduate training are more likely to form international ties than students who have not (OR = 4.78, p < 0.01). For faculty, no ego characteristics are significantly associated with the formation of international ties.

**Table 2:** Multilevel logistic regression of international ties on characteristics of egos, alters, ties, and networks.

	Model 1		Model 2		Model 3	
	Students	Faculty	Students	Faculty	Students	Faculty
Fixed Effects	(Odds Ratio)		(Odds Ratio)		(Odds Ratio)	
*Ego*						
Gender (1 = male)	1.363 (0.655)	0.731 (0.454)	2.403 (1.486)	0.132* (0.104)	2.000 (1.280))	0.097** (0.076)
Age (yrs) centered linear	0.961 (0.100)	0.971 (0.027)	0.903 (0.119)	0.976 (0.034)	0.236 (0.879)	0.994 (0.036)
Age square centered quadratic	1.017 (0.014)	1.003 (0.003)	1.022 (0.020)	0.999 (0.003)	1.024 (0.020)	0.998 (0.003)
Married (1 = yes)	0.210* (0.159)	1.249 (0.871)	0.120* (0.112)	1.520 (1.313)	0.142* (0.131)	1.765 (1.379)
No. of international conferences attended centered	1.132 (0.162)	1.076 (0.043)	1.032 (0.190)	1.048 (0.049)	0.985 (0.182)	1.065 (0.046)
Graduate training abroad (1 = yes)	4.781** (0.478)	3.895 (2.766)	9.408** (7.143)	1.253 (1.003)	8.220** (6.277)	1.707 (1.242)
*Alter*						
Gender (1 = male)			2.503 (1.271)	1.245 (0.687)	2.200 (1.156)	0.543 (0.352)
Length of time known (yrs) centered			1.234*** (0.070)	1.040 (0.033)	1.207** (0.073)	1.059 (0.037)
*Ties*						
Mode of communication^a^						
Conversational-only			0.003*** (0.003)	0.001*** (0.002)	0.004*** (0.005)	0.001*** (0.001)
Both			0.037*** (0.028)	0.012*** (0.010)	0.058** (0.048)	0.012*** (0.011)
Relationship^b^						
Mentor-mentee			0.764 (0.403)	0.071* (0.080)	0.620 (0.349)	0.119 (0.144)
Partner			45.785*** (42.415)	27.662** (29.153)	41.683*** (38.343)	17.366** (17.513)
Others			3.011 (1.914)	1.771 (0.910)	2.882 (1.812)	1.517 (0.785)
Intensity of Discussion (mins) centered			0.997* (0.001)	0.982*** (0.982)	0.997* (0.001)	0.980*** (0.005)
*Whole networks*						
Network size centered					1.142 (0.131)	0.848 (0.106)
Prop. of men centered					3.314 (4.872)	27.961* (40.817)
Prop. of nonconversational-only mode centered					6.055 (16.118)	0.949 (1.465)
Prop. of mentor-mentee relation centered					4.225 (5.701)	0.031* (0.052)
Mean length of time known (yrs) centered					1.106 (0.183)	0.862* (0.063)
Mean intensity of discussion (mins) centered					1.001 (0.002)	1.004** (0.002)
Random Effects	(Variance Component)		(Variance Component)		(Variance Component)	
Var(Intercept)	2.455 (0.994)	1.639 (0.773)	3.819 (1.925)	1.145 (0.783)	3.439 (1.846)	0.336 (0.482)
AIC	418.19	366.89	329.46	249.47	338.32	244.57
BIC	467.37	407.38	417.60	321.98	455.84	341.25
N	1011	424	989	415	989	415

***p < 0.001.

**p < 0.01.

*p < 0.05.

All models control for country.

Numbers in the parentheses are standard errors.

The reference group is non-conversational only mode.

The reference group is peer relationship.

In Model 2 the characteristics of alters and ties are added. For students, one alter characteristic and three tie characteristics are significantly associated with the formation of international ties. First, the length of time for which a student has known an alter is positively associated with a tie being international (OR = 1.23, p < 0.001). The longer a student has known an alter, the more likely it is that the tie is international. Second, the intensity of discussion is negatively associated with a tie being international (OR = 0.997, p < 0.001). The longer the discussion, the lower odds that the tie is international. This makes sense because the egos and international alters are less likely to be close to each other in terms of both time and geographic distance. Third, the mode of communication is associated with international ties. Using 1) conversational mode (e.g., face-to-face communication, phone, and Skype) and 2) both conversational and non-conversational mode is negatively associated with a tie being international, compared with strictly non-conversational mode communication (p < 0.001). That is, if the student uses only non-conversational mode to communicate with an alter, it is more likely that this tie is international. This finding is expected because of the potential difficulties in communicating via conversational mode due to time differences and geographic distance. Finally, the relationship of the tie is also associated with a tie being international. Compared with a peer relationship, a relationship related to partner is more likely to be international (OR = 45.785, p <0.0).

For faculty, the three tie characteristics aforementioned (i.e., intensity of discussion, mode of communication, and relationship of tie) are also significantly associated with the formation of international ties. First, similarly, the longer the discussion is, the lower the odds that the tie is international (OR = 0.98, p < 0.001). Second, likewise, if faculty use only a non-conversational mode to communicate with alters, it is more likely that this tie is international (p < 0.001). Third, compared with a peer relationship, a relationship related to mentor-mentee is less likely to be international (OR = 0.07, p < 0.05), whereas a relationship related to partner is more likely to be international (OR = 27.66, p < 0.05). It is worth noting that after adding the characteristics of alters and ties in Model 2, one ego characteristic–ego’s gender–which is not significant in Model 1 becomes significant (OR = 0.13, p < 0.05): male faculty are less likely to form international ties than female faculty.

Model 3 adds the characteristics of networks. For students, no network characteristics are significantly associated with cross-national ties. For faculty, four network characteristics have significant effects. First, the proportion of men in one’s network is positively associated with a cross-national tie (OR = 27.96, p < 0.05). Faculty who have a higher proportion of men in their networks are more likely to form international ties. Second, the proportion of mentor-mentee relationships in one’s network is negatively associated with international ties (OR = 60.03, p < 0.05). Faculty who have a higher proportion of mentor-mentee relationships in their network are less likely to form international ties. Third, the mean length of time for which faculty have known the alters in their network is negatively associated with the formation of international ties (OR = 0.86, p < 0.05). The longer mean length of time for which the faculty have known network members, the lower the odds that these ties are international. Last, the mean intensity of discussion between faculty and alters is positively associated with the formation of international ties (OR = 1.004, p < 0.01). The longer the mean length of discussion, the higher odds that these ties are international.

### International ties and research productivity

Finally, we turned to Poisson and negative binomial regressions to examine whether and how international ties influence research productivity, after controlling for ego-level characteristics, other network characteristics, and country. As shown in Table 3, manuscripts and papers in top journals among students are analyzed using Poisson regressions; all the others are analyzed using negative binomial regressions due to the over-dispersion of the count data. We will first present results of the students and then results of the faculty.

**Table 3:** Poisson and Negative Binomial regressions of research productivity on cross-national ties for students and faculty (N = 283).

	Manuscripts (IRR)		Papers (IRR)		Papers in top journals (IRR)	
	Students	Faculty	Students	Faculty	Students	Faculty
Intercept	0.036* (0.058)	0.006* (0.013)	0.000** (0.000)	0.007 (0.019)	0.000*** (0.000)	0.000*** (0.000)
*Whole networks*						
Prop. of international ties	2.947** (1.153)	1.415 (0.355)	8.463* (7.486)	1.074 (0.100)	1.891 (0.924)	3.013*** (0.920)
Prop. of men	0.620 (0.422)	2.303** (0.701)	4.028*** (0.176)	2.866** (1.110)	2.425 (1.699)	2.243* (0.849)
Prop. of nonconversational-only mode	0.534 (0.349)	1.195 (0.325)	0.377 (0.245)	1.539 (0.634)	0.079 (0.148)	0.922 (0.439)
Prop. of mentor-mentee relationship	1.441 (0.508)	0.533 (0.214)	1.176 (0.322)	0.572 (0.254)	2.192* (0.763)	1.412 (0.623)
Network size	0.982 (0.021)	0.984 (0.018)	0.956 (0.044)	0.990 (0.029)	1.049 (0.082)	1.003 (0.061)
Mean length of time known (yrs)	1.047** (0.017)	0.944*** (0.016)	0.911* (0.039)	0.926*** (0.015)	1.046 (0.113)	0.967 (0.025)
Mean intensity of discussion (mins)	0.9997 (0.001)	1.0003 (0.0002)	0.999 (0.0003)	0.999 (0.0002)	0.999 (0.001)	0.999 (0.0002)
*Ego*						
Time devoted to research (%)	0.991 (0.007)	1.003 (0.004)	0.997 (0.013)	0.997 (0.006)	0.994 (0.017)	1.006 (0.006)
No. of conferences attended	1.144*** (0.024)	1.012 (0.008)	1.221*** (0.031)	1.018* (0.008)	1.134*** (0.034)	1.003 (0.004)
Gender (1 = male)	1.344 (0.261)	1.352 (0.326)	0.974 (0.127)	1.147 (0.346)	1.560 (0.606)	1.039 (0.243)
Age (yrs)	1.243 (0.200)	1.246* (0.118)	2.724*** (0.691)	1.285 (0.171)	8.255** (6.068)	1.843** (0.333)
Age squared	0.997 (0.003)	0.998* (0.001)	0.984*** (0.004)	0.998 (0.001)	0.965** (0.013)	0.995** (0.002)
Married (1 = yes)	1.222 (0.167)	1.134 (0.360)	1.815** (0.316)	1.364 (0.343)	2.578* (0.218)	1.434 (0.506)
Graduate training abroad (1 = yes)	1.117 (0.198)	1.627 (0.426)	0.738 (0.176)	1.407 (0.407)	1.953** (0.446)	0.883 (0.211)
Alpha	-	0.204	0.207	0.371	-	0.272
Pseudo R^2^	0.17	0.11	0.16	0.12	0.25	0.19
N	201	82	201	82	201	82

***p < 0.001.

**p < 0.01.

*p < 0.05.

Manuscripts and papers in top journals among students are analyzed using Poisson regressions.

All the others are analyzed using negative binomial regressions.

Numbers in the parentheses are robust standard errors.

All regressions control for country.

For students, the proportion of international ties in one’s network is significantly and positively associated with the number of manuscripts (IRR = 2.947, p < 0.01) and the total number of published papers (IRR = 8.463, p < 0.05), but is not significantly associated with the number of papers published in top journals. Students who have a higher proportion of international ties in their networks wrote more manuscripts and published more papers in total, but the proportion of international ties made no difference in terms of publishing papers in top journals.

In addition to the proportion of international ties in one’s network, other network characteristics are also significantly associated with research productivity. First, the proportion of men in one’s network is positively associated with only the total number of papers published (IRR = 4.028, p < 0.001). Students who have a higher proportion of men in their network tend to publish more papers. Second, the proportion of mentor-mentee relationships is only positively associated with the number of papers published in top journals (IRR = 2.192, p < 0.05). Students who have a higher proportion of mentor-mentee relationships tend to publish more papers in top journals. Third, the mean length of time for which students have known alters in their total network is positively associated with the number of manuscripts (IRR = 1.047, p < 0.01), but negatively associated with the number of published papers (IRR = 0.221, p < 0.05). The longer time for which students knew their network members, the more manuscripts they wrote, but the fewer papers they published.

Characteristics of ego influence students’ research productivity as well. The number of conferences attended, student’s age, and marital status are significantly associated with research productivity. Students who attended more conferences tended to be more productive in terms of all three indicators. Second, married students tended to publish both more papers in total and in top journals. Third, the linear and quadratic terms of age are significant for the number of papers published in total and in top journals, suggesting a curvilinear relationship between age and the number of papers published in total and in top journals. As students’ age increased, the total number of papers published and the number of papers published in top journals first increased and then declined. Finally, experience in graduate training is positively associated with only the number of papers published in top journals. Students who have been abroad for graduate training published more papers in top journals ties than students who have never been abroad for graduate training.

For faculty, the proportion of international ties in one’s network is significantly and positively associated with only the number of papers published in top journals (IRR = 3.013p < 0.01). Faculty who have a higher proportion of international ties in their network published more papers in top journals. As for other network characteristics, the proportion of men in one’s network is positively associated with all three indicators of productivity. Faculty who have a higher proportion of men in their network tend to be more productive. Although previous studies [34, 35] identified no gender difference for collaboration among industrial-organizational psychologists and in economics, it is possible that male scientists are more connected than women scientists—the so called “old boy network”—in life sciences. Finally, the mean length of time for which faculty have known alters in their total network is negatively associated with the number of manuscripts (IRR = 0.944, p < 0.001) and the total number of papers published (IRR = 0.926, p < 0.001). The longer mean length of time for which faculty knew their network members, the fewer manuscripts they wrote and fewer papers they published. We speculate that this may be related to studies regarding strong and weak ties [20, 36, 37]. Studies show that weak ties are more important than strong ties to generate new ideas [36], which implies that strong ties may not be as beneficial as weak ties regarding a faculty’s productivity. This finding is also consistent with a study by Petersen [20] which found that collaborators frequently change over time in science based on co-authorship analysis.

In terms of the ego’s characteristics, similarly, the linear and quadratic terms of age are significant for 1) the number of manuscripts and 2) the number of papers published in top journals, suggesting a curvilinear relationship between age and the two outcomes. In addition, faculty who attended more conferences tended to publish more papers in total (IRR = 1.018, p < 0.05).

## Discussion

### Students

Our findings suggest that overseas graduate training can be a key factor for a graduate students’ ability to form international ties in these three countries. Living and studying abroad provides opportunities for students to build their professional networks with international scholars. This will likely lead to future collaboration. Jonkers and Tijssens [38] examined the relationship between research productivity and overseas experience among a sample of leading Chinese plant molecular life scientists who returned to their home country after receiving research experience abroad. Their results also supported that, in general, overseas research training experience has a positive impact on publication productivity. They attributed the benefit of the overseas experience to increasing skills and knowledge as well as connecting to professional networks internationally.

A faculty member in Singapore expressed the benefit of studying in the United States:

[I] think the U.S. has a lot to offer, for sure. The breadth of knowledge and the networks that the U.S. has built up are truly amazing. I think the working attitude, especially at the university, this drive for excellence and real impact is amazing in the U.S. [SM02]

A graduate student from Singapore realized the importance of graduate training overseas and wished for more opportunities:

I think there should be more exchange program, for graduate students. . . . I think you get to learn more about your research field and see how other universities, how they work. And also you have a better network of collaboration in future if you want. [SS07]

Governmental organizations appeared to understand the importance of studying overseas. For example, the China Scholarship Council in China provides funds for graduate students and postdocs to study overseas.

Some faculty believed that not only skills and knowledge but also diverse approaches to identifying scientific problems were gained through studying abroad. A Taiwanese faculty mentioned:

Somehow when I was in the United States I feel much, much more flexibility. The um, the school system and the professor. The professor give me more, much, much more um room, much, much more time for me to pursue my own goal. But, uh, my experience here, uh, the faculty members, they have their own topics or subjects and usually like the students to follow the topic or subject. [TM17]

Moreover, the network that they developed in host countries can benefit their research even after they returned to their home countries in the data set we examined. For example, we found that students who have a higher proportion of international ties in their networks tend to write more manuscripts and publish more papers, even after controlling for overseas graduate training, while graduate training itself has no effect on writing more manuscripts and publishing more papers. This suggests that research productivity based on these two indicators can be attributed to the social capital that students developed from the time they spent in their host countries, rather than the skills and knowledge that they developed when studying abroad, or the possibility that students who had overseas graduate training were simply better students in these three countries. However, we also found that international ties have no effect on publishing papers in top journals, whereas graduate training abroad is positively associated with publishing papers in top journals in this study. This seems to suggest that when it comes to research productivity based on quality (e.g., publications in top journals) rather than quantity (e.g., number of manuscripts and publications), the social capital that students obtained abroad does not matter; instead, what matters is the skills and knowledge that they obtained abroad or the quality of students themselves (that is, students who had overseas graduate training may have tended to be more talented and ambitious). A comment by a Taiwanese faculty member testified to this latter assertion:

usually… in Taiwan good students,… only get a master degree and then they go to USA to get their PhD degrees, just like me. I get a master’s degree in Taiwan and then I go to USA to get PhD degree.

In fact, this faculty member lamented the situation because it is very difficult to keep excellent students in Taiwan to collaborate with, although he further mentioned: “I think, they have their right to do their choice and sometimes if they go to USA, they go to a good laboratory and they find a good supervisor, I am happy for them too.” Other faculty members believed that studying overseas is important to becoming good scientists:

So I usually just tell my students you need to–if you have a chance- you need to go abroad to study. . . . everybody can buy facilities, money, hardware; but the, the strategy and attitude, and those things you cannot learn in a short term… you need to go to the [overseas]. [TM30]

Unfortunately, there is no way to test formally our speculations about the issue of causality and selection—the notion that students become more productive because they receive training abroad versus the notion that good students who are potentially more productive are more likely to go abroad—other than these interview data because we did not collect data on this question. This line of research could be pursued in future studies.

### Faculty

As for faculty, we found patterns that are distinctive from students. Faculty’s international ties have no effect on writing more manuscripts or publishing more papers, but do have an effect on publishing more papers in top journals in this study. This suggests that reaching out to international researchers can benefit scientists' research productivity in terms of quality rather than quantity in these three countries. Guerrero Bote et al.’s study [25] also found that papers with international collaborators tend to have higher citations. Conducting high quality research may require better equipment overseas or different expertise available only in foreign countries. Because the countries studied have relatively small research communities, they may have to reach beyond their borders to obtain the resources needed. The different patterns between students and faculty may reflect faculty and students’ differences in the use of international ties, or that there is less variance in ability and motivation among faculty than among students.

Of course, the international relationships that scientists cultivate may vary, as Ynalvez and Shrum [32] found in their study of professional networks among scientists in the Philippines. Those scientists who had graduate degrees from Japan tended to have strong, long-lasting relationships compared to those who studied in the U.S. and Australia. They speculated that this may be due to the curriculum difference—more apprenticeship in Japan—and mentoring style difference—more personal, frequent, and close mentoring.

We also found that graduate training abroad has no effect on research productivity for faculty. This is probably because the majority of faculty (80%) in these three countries have overseas graduate training. The faculty in Taiwan tended to be Taiwanese scientists who obtained their Ph.D.s from foreign institutions, primarily the U.S., U.K., Japan, or Australia, and came back to take positions at universities in Taiwan. In contrast, the faculty in Singapore are not necessarily from Singapore, where universities actively recruit foreign academics [39]. As such, all faculty we interviewed, except for three, obtained their doctorate from foreign countries such as Canada, Germany, the U.S., and India. All faculty in Japan, except for one, obtained their Ph.D.s from Japanese institutions.

These findings also have practical implications. Pouring resources into sending science graduate students to overseas universities will likely generate a high return on investment because these students tend to form international ties, and, as a result, they are more likely to publish more papers and write more manuscripts. Thus, these countries should feel encouraged to support exchange programs with overseas universities as well as to provide funds for students to study abroad.

However, sending students overseas is not the only way to foster international ties. Instead of sending students overseas, a university in Taiwan initiated a program to invite scholars from other countries to come to Taiwan. A faculty member explained how it works:

This time we will invite scholar from the U.S. [Another] time we will invite scholar from Japan too. So, when they come, these two faculty… need to sit together to discuss the service of the courses, and when they have decided, the […] syllabus goes to our faculty community to approve it. And after that the course will be offered to the student and the student can choose this as a course as they want to enroll in. I think, it's… not only benefit students, but also benefits the faculties because they can come here for a long time, like two or three weeks. We ask them, the invited scholars to, in addition to the teaching course, maybe stay in the lab to interact with the faculty and the lab members. [TM30, personal communication]

This is an innovative way to foster cross-national ties. He further mentioned how inviting foreign scholars could lead to future collaboration:

The student and faculty also benefit a lot. And also they can set up the future cooperation because they know each other they will say oh, this the field we are working on the same field so they can, even the week they stay, they can discuss the future research project tools. [TM30, personal communication]

## Limitations and conclusion

As with any study, the findings here should be interpreted with discretion. First, the sample size for the study is relatively small. Due to resource constraints, we were only able to collect data from approximately 100 scientists in each of the three countries. As such, the generalization of the study’s findings is limited although the findings may be applicable to countries with similar conditions. Second, we conducted surveys and interviews at the same time. If we had administered the surveys and followed up with interviews to ask specific questions regarding the results of the survey analysis, the study could have been richer. Nevertheless, the combination of both quantitative network surveys and qualitative interviews did provide different types of data and insights opposed to some previous studies that solely focused on either co-authorship data or social network analysis [18–20, 22]. Third, this study’s results were primarily derived from empirical data, not from a set of hypotheses based on a theoretical concept as is typical in social science studies. Although the study was rather explanatory in nature, we addressed a void in the literature and identified further research directions.

In conclusion, this study examined both graduate students and faculty in three East Asian countries in regard to their international ties and research productivity. The data indicate that graduate students in our sample who have received graduate training overseas are more likely to have cross-national ties in their egocentric social network, which has positive impacts on their productivity. Although many other factors influence a student’s productivity, interview data also indicate a series of benefits that students gain from studying overseas. Although graduate training overseas was not a factor for productivity among faculty, faculty with international ties are more likely to publish in top journals. Thus, fostering international ties for countries like Japan, Singapore, and Taiwan is considered important, and governments should support such efforts through funding and policies.

This study addressed faculty and graduate students’ professional networks using egocentric social network analysis. The networks that we examined were identified via interviews by asking with whom individuals discuss their research. These networks are not officially recorded elsewhere because these relationships may not appear in explicit co-authorship networks. However, as other studies have mentioned, it is useful to identify support networks for researchers as a type of scientific collaboration [2, 11]. This study sheds light on the relationships between these networks for productivity.

## Supporting information

S1 File Questionnaire for faculty. The questionnaire was used to collect data from faculty.
(DOC)

S2 File Questionnaire for students. The questionnaire was used to collect data from doctoral students.
(DOC)
