# Supplementary material for: Using egocentric analysis to investigate professional networks and productivity of graduate students and faculty in life sciences in Japan, Singapore, and Taiwan
Source: PLoS One. 2017 Oct 18;12(10):e0186608. doi: 10.1371/journal.pone.0186608 (PMC5646866; doi:10.1371/journal.pone.0186608)
Supplement: S1 File — The questionnaire was used to collect data from faculty. (DOC) [file pone.0186608.s001.doc]

**MOD-TRANSMISSION OF TACIT SKILLS IN EAST ASIAN**

**GRADUATE SCIENCE TRAINING PROGRAMS**

**(Japanese Advisers)**

**(*To be recorded using a Digital Voice Recorder)**

**RESPONDENT IDENTIFICATION**

1. NAME OF RESPONDENT (NOT TO BE CODED): ___________________________________________

2. RESPONDENT ID NUMBER: □ □ □

3. GENDER: □ MALE □ FEMALE

4. DATE (MM-DD-YY): □□-□□-□□

5. INTERVIEWER NAME: ________________________________________________

6. TIME INTERVIEW BEGAN: _______________ 7. TIME INTERVIEW ENDED: _____________

8. UNIVERSITY: _____________________________________________________________________

9. DEPARTMENT: ____________________________________________________________________

10. JOB TITILE: □ FULL PROFESSOR □ ASSOCIATE PROFESSOR

□ ASSISTANT PROFESSOR □ RESEARCH PROFESSOR

□ INSTRUCTOR □ POST-DOCTORAL FELLOW

□ RESEARCH ASSISTANT (Ph.D. degree holder in the case of Japan)

11. YEAR YOU JOINED THIS UNIVERSITY? ___ ___ (Note: *Joined the university as a faculty*)

12. HOW MANY YEARS DID YOU SPEND OUTSIDE JAPAN FOR GRADUATE TRAINING (*INCLUDE POST DOCTORAL TRAINING*)? ________

**EDUCATION**

| Degree | Field | Institution (University) | Country | Year Obtained |
| --- | --- | --- | --- | --- |
| 13. Ph.D./D.Sc. |  |  |  |  |
| 14. M.Sc./M.A. |  |  |  |  |
| 15. B.Sc./A.B. |  |  |  |  |

#### PERSONAL INFORMATION

16. What is your year of birth? □□□□

17. Are you married?

 1=married  2=not married

18. If married, what is your spouse’s main occupation?

 1=Farmer  2=K-12 Teacher/Educator  3=Civil Servant

 4=Nurse/Medical/Physician  5=Researcher/Professor/Scientist

 7=Business/Merchant/Shopkeeper  8=Houseperson  9=Other, specify ______

19. What is your father's main occupation?

 1=Laborer/Farmer/Worker  2=K-12 Teacher  3=Civil Servant

 4=Nurse/Medical/Physician  5=Researcher/Professor/Scientist

 7=Business/Merchant/Shopkeeper  8=Houseperson  9=Other, specify _____

20. Do you have children?  1=Yes  2=No

If YES:

21. How many children do you have? ________

22. How many are currently living/staying with you? ________

**PROFESSIONAL TIME ALLOCATION:** We'd like to know how your professional time is divided into the following **four** activities: research, teaching, administration, and community service. [CLARIFICATION: *WE MEAN YOUR ACTUAL TIME, NOT YOUR OFFICIAL TIME*]

23. About what percentage of your time is spent on research? ____%

24. About what percentage of your time is spent on teaching? ____%

25. About what percentage of your time is spent on administration? ____%

26. About what percentage of your time is spent on community service? ____%

**TOTAL = 100%**

**PERSONNEL**

27. Are there any people in this department that you supervise?  1=Yes  2=No

If YES: how many of each of the following people are you currently responsible for supervising?

28. ____ Professional scientists (e.g. professors, visiting professors, post docs)

29. ____ Research assistants/lab assistants /lab technicians

30. ____ Doctoral students

31. ____ Masteral students

32. ____ Undergraduate students

33. ____ Non-technical (administrative/secretarial/clerical) staff

34. Are there people in this department with whom you work closely?  1=Yes  2=No

If working closely with someone, how many of each of the following people in your own department do you work closely? By this I mean those with whom you currently discuss projects on a regular basis.

35. ____ Professional scientists (e.g. professors, visiting professors, post docs)

36. ____ Research assistants/lab assistants /lab technicians

37. ____ Doctoral students

38. ____ Masters students

39. ____ Undergraduate students

40. ____ Non-technical (administrative/secretarial/clerical) staff

**RESEARCH PROJECTS**

We are interested to know something about your main research projects and activities.

41. Currently, what is your main area of research and/or professional interest? Please give a couple of key words:

**Keywords:** _____________________________________________________________________________________

Could you please briefly give the title of each of your most important current projects? *EXPLAIN IF NECESSARY*.

We'd also like to know if each one is collaborative (that is, done in cooperation with someone in another department or organization) and where your collaborators are located. Please give up to a *MAXIMUM OF THREE PROJECTS ONLY*.

**42. FIRST PROJECT (IF ANY): Please give a couple of key words**

**Keywords:**

43. Is this a collaboration?  1=Yes  0=No

44. If YES, where are your collaborators located? (Check boxes for all that apply)

 1=in this location  2=in other locations in Japan

 3=in Australia  4=in the United Kingdom

 5=in the United States  6=in another country, please specify: _________________________________

45. What month and year did this project start? Month: __________ Year: __________

**46. SECOND PROJECT (IF ANY): Please give a couple of key words**

**Keywords:**

47. Is this a collaboration?  1=Yes  0=No

48. If YES, where are your collaborators located? (Check boxes for all that apply)

 1=in this location  2=in other locations in Japan

 3=in Australia  4=in the United Kingdom

 5=in the United States  6=in another country, please specify: __________________________

49. What month and year did this project start? Month: __________ Year: __________

**50. THIRD PROJECT (IF ANY): Please give a couple of key words**

**Keywords:**

51. Is this a collaboration?  1=Yes  0=No

52. If YES, where are your collaborators located? (Check boxes for all that apply)

 1=in this location  2=in other locations in Japan

 3=in Australia  4=in the United Kingdom

 5=in the United States  6=in another country, please specify: __________________________

53. What month and year did this project start? Month: __________ Year: __________

**YOUR PROJECTS IN GENERAL:**

54. Total number of research projects you are currently involved in: _________

55. Total number of research projects you are currently directing (role as principal investigator): _________

56. Since 2006, how many collaborative projects have you been involved in? _________

**SUPPORT NETWORK**

Now I'd like to ask about a few specific people, the people who have similar interests or work on the same kinds of things that you do. Here, include anyone you talk to, go to for advice, or anyone who comes to you for advice. In other words, just tell me those that are the most important for your own work.

**PROBLEMS ENCOUNTERED IN CURRENT RESEARCH:**

If each of the following is a problem for you in any of your current research, please rate this on a scale of 1 to 5 with 1 indicating not a problem and 5 indicating a major problem:

|  | Not a problem |  |  |  | Major problem |
| --- | --- | --- | --- | --- | --- |
| 57. Contacting people when they are needed | 1 | 2 | 3 | 4 | 5 |
| 58. Coordinating schedules | 1 | 2 | 3 | 4 | 5 |
| 59. Length of time to get things done | 1 | 2 | 3 | 4 | 5 |
| 60. Transmitting information | 1 | 2 | 3 | 4 | 5 |
| 61. Getting others to see your point | 1 | 2 | 3 | 4 | 5 |
| 62. Data management and security of information | 1 | 2 | 3 | 4 | 5 |
| 63. Resolving conflicts | 1 | 2 | 3 | 4 | 5 |
| 64. Decisions on a division of work | 1 | 2 | 3 | 4 | 5 |
| 65. Heavy administrative demands | 1 | 2 | 3 | 4 | 5 |
| 66. Heavy teaching load | 1 | 2 | 3 | 4 | 5 |

**RESOURCES FOR RESEARCH**

67. Are the equipment needed to do your research available?  1=Yes  0=No

68. …how about the supplies and resources you need?  1=Yes  0=No

69. …how about the professional staff (e.g. biostatistician, copy editor) you need?  1=Yes  0=No

70. …how about the support staff (lab tech., secretarial, clerical) you need?  1=Yes  0=No

**PROFESSIONAL ACTIVITIES**

71. **In answering the following questions, please think as far back as 2008.**

72. Are you a member of any professional organizations?  1=Yes  0=No

73. Have you served on the editorial board of a journal?  1=Yes  0=No

74. Have you held a position in a professional organization?  1=Yes  0=No

75. Have you been a member of a government committee or advisory group?  1=Yes  0=No

76. Have you served as a consultant within your professional field?  1=Yes  0=No

77. Have you been to any training courses during this period?  1=Yes  0=No

78. In one way or another, have you reviewed a manuscript for a journal?  1=Yes  0=No

**RESEARCH TIME**

79. In a typical week, how many hours do you spend doing research? _____

80. In a typical week, how many hours do you spend doing lab work or running experiments? _____

81. In a typical week, how many hours do you supervise or hold consultations with your doctoral students?* _____

82. In a typical week, how many hours do you spend writing papers? _____

**REPORTING FOR WORK, BREAK TIME, VACATIONS/HOLIDAYS**

83. Typically, what time do you come to office? _____

84. Typically, what time do you go home? _____

85. Once home, do you come back to office after dinner?  1=Yes  0=No

86. Typically, what time do you go for tea and/or coffee breaks? _____

87. On average, how long would short break (tea and/or coffee) times be? _____ (record in minutes)

88a. Typically, what time do you go for lunch? _____ (time)

88b. On average, how long do you go out for lunch? _____ (record in minutes)

89. In a year, about how many days do you take off for holidays/vacations? _____ (record in days)

### RESEARCH SKILLS

I would like to know about the three most important research skills for you to do your work. I would also like to know how you acquired these skills.

| Research Skills | How long did it take you to learn this skill? | Who taught you? |
| --- | --- | --- |
| 90. |  |  |
| 91. |  |  |
| 92. |  |  |

### RESEARCH PRODUCTIVITY

93. Since 2008, have you presented papers and/or posters in conferences?  1=Yes  0=No

**If yes,**

94. How many did you present at domestic conferences (non-internationally sponsored)? _______

95. How many did you present at internationally sponsored conferences? _______

96. Since 2008, have you published a paper?  1=Yes  0=No

**If yes,**

97. How many are articles in international journals? _______

98. How many are articles in national/domestic journals? _______

99. How many are articles in top journals? _______

100. How many are chapters in books? _______

101. Since 2008, have you received any science/research awards?  1=Yes  0=No

**If yes,**

102. How many are national science/research awards? _______

103. How many are international science/research awards? _______

104. Since 2008, have you received any research grants?  1=Yes  0=No

**If yes,**

105. How many are funded by your university? _______

106. How many are nationally funded grants? _______

107. How many are internationally funded grants? _______

108. Since 2008, how many patents have you generated? _______

109. In the last 12 months, how many manuscripts (published/unpublished) have you written? _______

**CONFERENCES**

110. Since 2008, have you attended any conferences?  1=Yes  0=No

**If yes,**

111. How many conferences have you attended? _______

112. How many conferences have you attended within Japan? _______

113. How many conferences have you attended *outside Japan but within Asia*? _______

114. How many conferences have you attended *outside Asia*? _______

**IMPROVING YOUR GRADUATE SCIENCE PROGRAM**

Think of the following 9 items, how would you rank their importance for improving graduate science programs in Japan? Give a number from 1 to 9, where 1 is most important and 9 is least important. Do not use any number more than once. (**Note: You may ask respondent to fill out the numbers himself/herself.**)

115. Internationally competitive salaries for professors/researchers ____

116. Seed money for start-up research ____

117. Electronic data bases/library/equipment budget ____

118. Budget for conference travel ____

119. Budget for communication ____

120. Training budget for technicians and research personnel ____

121. Assistantship/scholarships for graduate students ____

122. Recruitment of international graduate students ____

123. Recruitment of professors from abroad ____

**COMPUTER USE**

AT HOME/APARTMENT/DORM (**NOT IN THE LAB**):

124. In a typical week, about how many hours do you use a computer for your research? _____

125. How many computers do you have at home? _____

126. How many people use a computer at home? _____

127. How many of home computers are Internet connected? _____

AT THE LABORATORY:

128. In a typical week, about how many hours do you use a computer for your research? _____

129. How many computers do you have at the lab? _____

130. How many people use a computer at the lab? _____

131. How many of these lab computers are Internet connected? _____

**CELLPHONE USE**

132. Do you have a cell phone?  1=Yes  0=No

**If yes,**

133. In a day, about how many hours do you use your cell phone for research? _____

134. … about how many calls related to research do you receive? _____

135. … about how many calls related to research do you make? _____

136. How many years have you had a cell phone? _____

# WITH YOUR CELLPHONE, HAVE YOU EVER DISCUSSED RESEARCH WITH

137. …graduate students?  1=Yes  0=No

138. …another professor*?  1=Yes  0=No

139. …my major professor/mentor?  1=Yes  0=No

140. …a colleague in another location in Japan?  1=Yes  0=No

141. …a colleague in Asia but outside Japan?  1=Yes  0=No

142. …a colleague in the developed countries (e.g. U.S., U.K)?  1=Yes  0=No

143. …a colleague in the developing countries (e.g. Indonesia, Philippines)?  1=Yes  0=No

144. …funding agencies?  1=Yes  0=No

**EMAIL USE**

FOR THESE NEXT QUESTIONS, THINK ABOUT YOUR **TYPICAL WEEK**:

145. How many hours do you spend receiving and sending email? _________

146. How many email messages do you send?

1 = Less than one a week

2 = Less than two per day

3 = Three to ten per day

4 = More than ten daily

147. How many of these messages you send are related to your research?

1 = Less than one a week

2 = Less than two per day

3 = Three to ten per day

4 = More than ten daily

148. How many email messages (EXCLUDE JUNK MAIL) do you receive?

1 = Less than one a week

2 = Less than two per day

3 = Three to 10 per day

4 = More than ten daily

149. How many of these messages you receive are related to your research?

1 = Less than one a week

2 = Less than two per day

3 = Three to 10 per day

4 = More than ten daily

# HAVE YOU EVER DONE THE FOLLOWING ON EMAIL?

150. Discussed research with graduate students  1=Yes  0=No

151. Discussed research with another professor*  1=Yes  0=No

152. Discussed research with my major professor/mentor  1=Yes  0=No

153. Discussed research with a colleague in another location in Japan  1=Yes  0=No

154. Discussed research with a colleague in Asia but outside the Japan  1=Yes  0=No

155. Discussed research with a colleague in the developed countries (e.g. U.S., U.K)  1=Yes  0=No

156. Discussed research with a colleague in the developing countries (e.g. Indonesia, Philippines) 1=Yes  0=No

157. Started a professional relationship with someone you met through email  1=Yes  0=No

158. Reviewed a manuscript for journals  1=Yes  0=No

159. Discussed proposals with funding agencies  1=Yes  0=No

160. Submitted manuscripts for journal  1=Yes  0=No

**WEB USE**

161. In a typical week, how many hours do you spend using the web? __________

162. In a typical week, how many hours are spent on matters related to your research? __________

WHICH OF THE FOLLOWING HAVE YOU DONE ON-LINE?

163.…purchased a product or service for your research  1=Yes  0=No

164.…created a research project homepage  1=Yes  0=No

165.…conducted an information search  1=Yes  0=No

166.…used scientific research data  1=Yes  0=No

167.…collaborated on a scientific project  1=Yes  0=No

168.…conducted Internet-based conference or meeting for your research  1=Yes  0=No

169.…consulted another researcher through on-line telephony (e.g. Skype)  1=Yes  0=No

170.…found and examined reference materials (e.g. dictionary, encyclopedia)  1=Yes  0=No

171.…accessed research reports or scientific papers  1=Yes  0=No

172.…downloaded research software  1=Yes  0=No

173.…used an on-line data analysis software  1=Yes  0=No

**MENTORING PROBLEMS AND WHAT STUDENTS DO**

The following is a list of problems professors report in mentoring graduate students. Based on your experience, is this a problem for you? Please evaluate each of these problem areas using the following scale:

1 = not a problem, 5 = major problem.

| Problems/Concerns | | Rating  NP MP | | | | | | | | | |
| --- | --- | --- | --- | --- | --- | --- | --- | --- | --- | --- | --- |
| 174. Student not coming to the laboratory | | 1 | | 2 | | 3 | | 4 | | 5 | |
| 175. Student not submitting assignments and reports on time | | 1 | | 2 | | 3 | | 4 | | 5 | |
| 176. Student uses computer and Internet for non-research purposes | | 1 | | 2 | | 3 | | 4 | | 5 | |
| 177. Student not maintaining organized laboratory notes and research data | | 1 | | 2 | | 3 | | 4 | | 5 | |
| 178. Student disrespectful to other students | | 1 | | 2 | | 3 | | 4 | | 5 | |
| 179. Student disrespectful to staff and professor | | 1 | | 2 | | 3 | | 4 | | 5 | |
| 180. Instructing students on what to do | | 1 | | 2 | | 3 | | 4 | | 5 | |
| 181. Students does not ask permission from staff and professor | | 1 | | 2 | | 3 | | 4 | | 5 | |
| 182. Student fighting with another student | | 1 | | 2 | | 3 | | 4 | | 5 | |
| 183. Student does not consult with staff professor | | 1 | | 2 | | 3 | | 4 | | 5 | |
| 184. Student not attending lab meetings | | 1 | | 2 | | 3 | | 4 | | 5 | |
| 185. Student not providing updates on research work | | 1 | | 2 | | 3 | | 4 | | 5 | |
| 186. Student has difficulty communicating orally and written | | 1 | | 2 | | 3 | | 4 | | 5 | |
| 187. Student leaves equipment and experimental set-up unattended | | 1 | | 2 | | 3 | | 4 | | 5 | |
| Please rate the following practices in terms of their frequency using the following scale:  1 = never, 2 = rarely, 3 = often, 4 = very often. Since 2008, how frequently you have done the following: | | | | | | | | | | | |
|  |  | | N | |  | |  | | Vo | |  |
|  | 188. discuss career aspirations and plans of students | | 1 | | 2 | | 3 | | 4 | |  |
|  | 189. monitor students’ work progress | | 1 | | 2 | | 3 | | 4 | |  |
|  | 190. discuss students’ concerns and problems about their research | | 1 | | 2 | | 3 | | 4 | |  |
|  | 191. discuss personal and/or family problems of students | | 1 | | 2 | | 3 | | 4 | |  |
|  | 192. co-author research paper or book chapter with students | | 1 | | 2 | | 3 | | 4 | |  |
|  | 193. co-direct research project with students | | 1 | | 2 | | 3 | | 4 | |  |
|  | 194. analyze data and perform calculations side-by-side with students | | 1 | | 2 | | 3 | | 4 | |  |
|  | 195. run experiments side-by-side with students | | 1 | | 2 | | 3 | | 4 | |  |
|  | 196. review students for general and comprehensive exams | | 1 | | 2 | | 3 | | 4 | |  |
|  | 197. help students draft job application letters | | 1 | | 2 | | 3 | | 4 | |  |
|  | 198. help students draft their curriculum vitae | | 1 | | 2 | | 3 | | 4 | |  |
|  | 199. help students prepare for a job talk or presentation | | 1 | | 2 | | 3 | | 4 | |  |
|  | 200. help students search for job positions and announcements | | 1 | | 2 | | 3 | | 4 | |  |
|  | 201. socialize students to members of the professional community | | 1 | | 2 | | 3 | | 4 | |  |
|  | 202. give feedback on students’ research and performance | | 1 | | 2 | | 3 | | 4 | |  |

**GRADUATE TRAINING PRACTICES**

| Please rate the following practices in terms of their frequency using the following scale:  1 = never, 2 = rarely, 3 = often, 4 = very often. Since 2008, how frequently have your graduate students done the following: | Rating | | | |
| --- | --- | --- | --- | --- |
| 1. Present research in departmental seminars | 1 | 2 | 3 | 4 |
| 1. Present research in conferences (national, regional, international) | 1 | 2 | 3 | 4 |
| 1. Participate in research competitions (national, regional, international) | 1 | 2 | 3 | 4 |
| 1. Attend trainings to enhance research skills and techniques | 1 | 2 | 3 | 4 |
| 1. Organize professional meetings and conferences | 1 | 2 | 3 | 4 |
| 1. Perform data analyses | 1 | 2 | 3 | 4 |
| 1. Write and submit grant proposals | 1 | 2 | 3 | 4 |
| 1. Take the lead in a research lab meeting | 1 | 2 | 3 | 4 |
| 1. Review and comment on manuscripts which you were/are reviewing | 1 | 2 | 3 | 4 |
| 1. Write papers for submission to scholarly journals | 1 | 2 | 3 | 4 |
| 1. Draft letters to the editor for submission of manuscripts | 1 | 2 | 3 | 4 |
| 1. Draft responses to reviewers for revised and resubmitted manuscripts | 1 | 2 | 3 | 4 |
| 1. Visit other laboratories to learn skills and techniques | 1 | 2 | 3 | 4 |
| 1. Draft operating manuals for lab instruments and equipment | 1 | 2 | 3 | 4 |
| 1. Have senior students help junior students in their research | 1 | 2 | 3 | 4 |
| 1. Review and comment on reports and papers produced by the laboratory | 1 | 2 | 3 | 4 |
| 1. Review and critique recently published leading research articles | 1 | 2 | 3 | 4 |

**INTERACTION WITH GRADUATE STUDENTS**

The following is a list of bi-polar adjectives that describes advisers’ interactions with their graduate students. Please rate each pair of adjectives based on your own personal experiences as an adviser.

| *220. Face-to-face* | -3 | -2 | -1 | 0 | 1 | 2 | 3 | *Technology-mediated* |
| --- | --- | --- | --- | --- | --- | --- | --- | --- |
| *221. Formal/Impersonal* | -3 | -2 | -1 | 0 | 1 | 2 | 3 | *Informal/Personal* |
| *222. Frequent* | -3 | -2 | -1 | 0 | 1 | 2 | 3 | *Seldom* |
| *223. One-way* | -3 | -2 | -1 | 0 | 1 | 2 | 3 | *Two-way* |
| *224. To look forward to* | -3 | -2 | -1 | 0 | 1 | 2 | 3 | *To avoid* |
| *225. Exciting* | -3 | -2 | -1 | 0 | 1 | 2 | 3 | *Boring* |
| *226. Quick talks* | -3 | -2 | -1 | 0 | 1 | 2 | 3 | *Long discussions* |
| *227. Structured/hierarchical* | -3 | -2 | -1 | 0 | 1 | 2 | 3 | *Unstructured/non-hierarchical* |
| *228. Supportive* | -3 | -2 | -1 | 0 | 1 | 2 | 3 | *Adversarial* |
| *229. Friendly* | -3 | -2 | -1 | 0 | 1 | 2 | 3 | *Unfriendly* |
| *230. Considerate* | -3 | -2 | -1 | 0 | 1 | 2 | 3 | *Inconsiderate* |
| *231. General/Broad* | -3 | -2 | -1 | 0 | 1 | 2 | 3 | *Specifics/Detailed* |
| *232. Planned* | -3 | -2 | -1 | 0 | 1 | 2 | 3 | *Unplanned* |

**WORKING IN THE RESEARCH LABORATORY**

The following is a list of bi-polar adjectives that describes work environment in a research laboratory. Please rate each pair of adjectives based on your own personal experiences in such a setting.

| *233. Many things to learn* | -3 | -2 | -1 | 0 | 1 | 2 | 3 | *Nothing to learn* |
| --- | --- | --- | --- | --- | --- | --- | --- | --- |
| *234.Many things to do* | -3 | -2 | -1 | 0 | 1 | 2 | 3 | *Nothing to do* |
| *235. Competitive* | -3 | -2 | -1 | 0 | 1 | 2 | 3 | *Cooperative* |
| *236. To look forward to* | -3 | -2 | -1 | 0 | 1 | 2 | 3 | *To avoid* |
| *237. Exciting* | -3 | -2 | -1 | 0 | 1 | 2 | 3 | *Boring* |
| *238. Structured/hierarchical* | -3 | -2 | -1 | 0 | 1 | 2 | 3 | *Unstructured/non-hierarchical* |
| *239. Busy* | -3 | -2 | -1 | 0 | 1 | 2 | 3 | *Relaxed* |
| *240. Supportive* | -3 | -2 | -1 | 0 | 1 | 2 | 3 | *Adversarial* |
| *241. Friendly* | -3 | -2 | -1 | 0 | 1 | 2 | 3 | *Unfriendly* |
| *242. Open communication* | -3 | -2 | -1 | 0 | 1 | 2 | 3 | *Close communication* |
| *243. Authoritarian* | -3 | -2 | -1 | 0 | 1 | 2 | 3 | *Democratic* |
| *244. Has privacy* | -3 | -2 | -1 | 0 | 1 | 2 | 3 | *Has no privacy* |
| *245. Low supervision* | -3 | -2 | -1 | 0 | 1 | 2 | 3 | *High supervision* |

**OTHER INFORMATION**

246. What is your nationality?

 (1) Japanese  (2) Singaporean  (3) Taiwanese  (4) Chinese

 (5) Korean  (6) others, please specify: ______________________

247. In a typical week, how often do you go to church for mass or religious services?

 (1) Never

 (2) Once a week

 (3) Two to three times a week

 (4) Four to five times a week

 (5) Everyday

**Thanks very much for your time.**

**INTERVIEWER’S OBSERVATION**

248. What was respondent’s initial attitude about being interviewed?

 (1) Very interested

 (2) Somewhat interested

 (3) Indifferent

 (4) Somewhat reluctant

 (5) Very reluctant

 (6) Hard to tell

249. Was anyone else present during the interview?

 (1) Yes  (2) No

250. How open and forthcoming do you think respondent was about his/her feelings?

 (1) Very open

 (2) Held back somewhat

 (3) Held back a great deal

251. Did you observe any signs of tension or stress in respondent’s behavior?

 (1) Yes  (2) No

252. Where was the interview held?

 (1) Private setting (respondent’s own room and not shared with others)

 (2) Semi-private setting (respondent’s own room but shared with others)

 (3) Public setting (library, cafeteria, lobby; laboratory)
