# Supplementary material for: Using egocentric analysis to investigate professional networks and productivity of graduate students and faculty in life sciences in Japan, Singapore, and Taiwan
Source: PLoS One. 2017 Oct 18;12(10):e0186608. doi: 10.1371/journal.pone.0186608 (PMC5646866; doi:10.1371/journal.pone.0186608)
Supplement: S2 File — The questionnaire was used to collect data from doctoral students. (DOC) [file pone.0186608.s002.doc]

**MOD-TRANSMISSION OF TACIT SKILLS IN EAST ASIAN**

**GRADUATE SCIENCE TRAINING PROGRAMS**

**(For Japanese Doctoral Students)**

**(*To be recorded using a Digital Voice Recorder)**

**RESPONDENT IDENTIFICATION**

1. NAME OF RESPONDENT (NOT TO BE CODED): ___________________________________________

2. RESPONDENT ID NUMBER: □ □ □

3. GENDER: □ MALE □ FEMALE

4. DATE (MM-DD-YY): □□-□□-□□

5. INTERVIEWER NAME: ________________________________________________

6. TIME INTERVIEW BEGAN: _______________ 7. TIME INTERVIEW ENDED: _____________

8. UNIVERSITY: _____________________________________________________________________

9. DEPARTMENT: ____________________________________________________________________

10. JOB TITILE: □ First year doctoral student □ Third year doctoral student

□ Second year doctoral student □ Fourth year or plus doctoral student

11. YEAR YOU JOINED THIS UNIVERSITY? ___ ___ (Note: *Joined the university as a doctoral student*)

12. HOW MANY YEARS DID YOU SPEND OUTSIDE JAPAN FOR GRADUATE TRAINING (INCLUDE POST DOCTORAL TRAINING)? ________

**EDUCATION**

| Degree | Field | Institution (University) | Country | Year Expected |
| --- | --- | --- | --- | --- |
| 13. Ph.D./D.Sc. |  |  |  |  |
| 14. M.Sc./M.A. |  |  |  |  |
| 15. B.Sc./A.B. |  |  |  |  |

#### PERSONAL INFORMATION

16. What is your year of birth? □□□□

17. Are you married?

 1=married  2=not married

18. If married, what is your spouse’s main occupation?

 1=Farmer  2=K-12 Teacher/Educator  3=Civil Servant

 4=Nurse/Medical/Physician  5=Researcher/Professor/Scientist

 7=Business/Merchant/Shopkeeper  8=Houseperson  9=Other, specify ______

19. What is your father's main occupation?

 1=Laborer/Farmer/Worker  2=K-12 Teacher  3=Civil Servant

 4=Nurse/Medical/Physician  5=Researcher/Professor/Scientist

 7=Business/Merchant/Shopkeeper  8=Houseperson  9=Other, specify _____

20. Do you have children?  1=Yes  2=No

If YES:

21. How many children do you have? ________

22. How many are currently living/staying with you? ________

**PROFESSIONAL TIME ALLOCATION:** We'd like to know how your professional time is divided into the following **four** activities: research, teaching, administration, and community service. [CLARIFICATION: *WE MEAN YOUR ACTUAL TIME, NOT YOUR OFFICIAL TIME*]

23. About what percentage of your time is spent on research? ____%

24. About what percentage of your time is spent on teaching? ____%

25. About what percentage of your time is spent on administration? ____%

26. About what percentage of your time is spent on community service? ____%

**TOTAL = 100%**

**PERSONNEL**

27. Are there any people in this department that you supervise?  1=Yes  2=No

If YES: how many of each of the following people are you currently responsible for supervising?

28. ____ Research assistants/lab assistants /lab technicians

29. ____ Doctoral students

30. ____ Masteral students

31. ____ Undergraduate students

32. ____ Non-technical (administrative/secretarial/clerical) staff

33. Are there people in this department with whom you work closely?  1=Yes  2=No

If working closely with someone, how many of each of the following people in your own department do you work closely? By this I mean those with whom you currently discuss projects on a regular basis.

34. ____ Professional scientists (e.g. professors, post docs)

35. ____ Research assistants/lab assistants /lab technicians

36. ____ Doctoral students

37. ____ Masters students

38. ____ Undergraduate students

39. ____ Non-technical (administrative/secretarial/clerical) staff

**RESEARCH PROJECTS**

We are interested to know something about your main research projects and activities.

40. Currently, what is your main area of research and/or professional interest? **Please give a couple of key words:** **KEYWORDS:** __________________________________________________________________________________

Could you please briefly give the title of each of your most important current projects? *EXPLAIN IF NECESSARY*.

We'd also like to know if each one is collaborative (that is, done in cooperation with someone in another department or organization) and where your collaborators are located. Please give up to a *MAXIMUM OF THREE PROJECTS ONLY*.

**41. FIRST PROJECT (IF ANY): Please give a couple of key words**

**KEYWORDS:**

42. Is this a collaboration?  1=Yes  0=No

43 If YES, where are your collaborators located? (Check boxes for all that apply)

 1=in this location  2=in other locations in Japan

 3=in Australia  4=in the United Kingdom

 5=in the United States  6=in another country, please specify: _________________________________

44. What month and year did this project start? Month: __________ Year: __________

**45. SECOND PROJECT (IF ANY): Please give a couple of key words**

**KEYWORDS:**

46. Is this a collaboration?  1=Yes  0=No

47. If YES, where are your collaborators located? (Check boxes for all that apply)

 1=in this location  2=in other locations in Japan

 3=in Australia  4=in the United Kingdom

 5=in the United States  6=in another country, please specify: __________________________

48. What month and year did this project start? Month: __________ Year: __________

**49. THIRD PROJECT (IF ANY): Please give a couple of key words**

**KEYWORDS:**

50. Is this a collaboration?  1=Yes  0=No

51. If YES, where are your collaborators located? (Check boxes for all that apply)

 1=in this location  2=in other locations in Japan

 3=in Australia  4=in the United Kingdom

 5=in the United States  6=in another country, please specify: __________________________

52. What month and year did this project start? Month: __________ Year: __________

**YOUR PROJECTS IN GENERAL:**

53. Total number of research projects you are currently involved in: _________

54. Total number of research projects you are currently directing (role as principal investigator): _________

55. Since 2006, how many collaborative projects have you been involved in? _________

**SUPPORT NETWORK**

Now I'd like to ask about a few specific people, the people who have similar interests or work on the same kinds of things that you do. Here, include anyone you talk to, go to for advice, or anyone who comes to you for advice. In other words, just tell me those that are the most important for your own work.

**PROBLEMS ENCOUNTERED IN CURRENT RESEARCH:**

If each of the following is a problem for you in any of your current research, please rate this on a scale of 1 to 5 with 1 indicating not a problem and 5 indicating a major problem:

|  | Not a problem |  |  |  | Major problem |
| --- | --- | --- | --- | --- | --- |
| 56. Contacting people when they are needed | 1 | 2 | 3 | 4 | 5 |
| 57. Coordinating schedules | 1 | 2 | 3 | 4 | 5 |
| 58. Length of time to get things done | 1 | 2 | 3 | 4 | 5 |
| 59. Transmitting information | 1 | 2 | 3 | 4 | 5 |
| 60. Getting others to see your point | 1 | 2 | 3 | 4 | 5 |
| 61. Data management and security of information | 1 | 2 | 3 | 4 | 5 |
| 62. Resolving conflicts | 1 | 2 | 3 | 4 | 5 |
| 63. Decisions on a division of work | 1 | 2 | 3 | 4 | 5 |
| 64. Heavy administrative demands | 1 | 2 | 3 | 4 | 5 |
| 65. Heavy teaching load | 1 | 2 | 3 | 4 | 5 |

**RESOURCES FOR RESEARCH**

66. Are the equipment needed to do your research available?  1=Yes  0=No

67.…how about the supplies and resources you need?  1=Yes  0=No

68. …how about the professional staff (e.g. biostatistician, copy editor) you need?  1=Yes  0=No

69. …how about the support staff (lab tech., secretarial, clerical) you need?  1=Yes  0=No

**PROFESSIONAL ACTIVITIES**

**In answering the following questions, please think as far back as 2008.**

70. Are you a member of any professional organizations?  1=Yes  0=No

71. Have you served on the editorial board of a journal?  1=Yes  0=No

72. Have you held a position in a professional organization?  1=Yes  0=No

73. Have you been a member of a government committee or advisory group?  1=Yes  0=No

74. Have you served as a consultant within your professional field?  1=Yes  0=No

75. Have you been to any training courses during this period?  1=Yes  0=No

76. In one way or another, have you reviewed a manuscript for a journal?  1=Yes  0=No

**RESEARCH TIME**

77. In a typical week, how many hours do you spend doing research? ______

78. In a typical week, how many hours do you spend doing lab work or running experiments? ______

79. In a typical week, how many hours do you consult with or supervised by your professor?* ______

80. In a typical week, how many hours do you spend writing papers? ______

**REPORTING FOR WORK, BREAK TIME, VACATIONS/HOLIDAYS**

81. Typically, what time do you come to office? _____

82. Typically, what time do you go home? _____

83. Once home, do you come back to office after dinner?  1=Yes  0=No

84. Typically, what time do you go for tea and/or coffee breaks? _____

85. On average, how long would short break (tea and/or coffee) times be? _____ (record number of minutes)

86. Typically, what time do you go for lunch? _____ (time)

87. On average, how long do you go out for lunch? _____ (record number of minutes)

88. In a year, about how many days do you take off for holidays/vacations? _____ (record in days)

### RESEARCH SKILLS

I would like to know about the three most important research skills for you to do your work. I would also like to know how you acquired these skills.

| Research Skills | How long did it take you to learn this skill? | Who taught you? |
| --- | --- | --- |
| 89. |  |  |
| 90. |  |  |
| 91. |  |  |

### RESEARCH PRODUCTIVITY

92. Since 2008, have you presented papers and/or posters in conferences?  1=Yes  0=No

**If yes,**

93. How many did you present at domestic conferences (non-internationally sponsored)? _______

94. How many did you present at internationally sponsored conferences? _______

95. Since 2008, have you published a paper?  1=Yes  0=No

**If yes,**

96. How many are articles in international journals? _______

97. How many are articles in national/domestic journals? _______

98. How many are articles in top journals? _______

99. How many are chapters in books? _______

100. Since 2008, have you received any science/research awards?  1=Yes  0=No

**If yes,**

101. How many are national science/research awards? _______

102. How many are international science/research awards _______

103. Since 2008, have you received any research grants?  1=Yes  0=No

**If yes,**

104. How many are funded by your university? _______

105. How many are nationally funded grants? _______

106. How many are internationally funded grants? _______

107. Since 2008, how many patents have you generated? _______

108. In the last 12 months, how many manuscripts (published or unpublished) have you written? _______

**CONFERENCES**

109. Since 2008, have you attended any conferences?  1=Yes  0=No

**If yes,**

110. How many conferences have you attended? _______

111. How many conferences have you attended within Japan? _______

112. How many conferences have you attended *outside Japan but within Asia*? _______

113. How many conferences have you attended *outside Asia*? _______

**IMPROVING YOUR GRADUATE SCIENCE PROGRAM**

Think of the following 9 items, how would you rank their importance for improving graduate science programs in Japan? Give a number from 1 to 9, where 1 is most important and 9 is least important. Do not use any number more than once. (**Note: You may ask respondent to fill out the numbers himself/herself.**)

114. Internationally competitive salaries for professors/researchers/scientists ____

115. Seed money for start-up research ____

116. Electronic data bases/library/equipment budget ____

117. Budget for conference travel ____

118. Budget for communication ____

119. Training budget for technicians and research personnel ____

120. Assistantship/scholarships for graduate students ____

121. Recruitment of international graduate students ____

122. Recruitment of professors from abroad ____

**COMPUTER USE**

AT HOME/APARTMENT/DORM (**NOT IN THE LAB**):

123. In a typical week, about how many hours do you use a computer for your research? _____

124. How many computers do you have at home _____

125. How many people use a computer at home? _____

126. How many of home computers are Internet connected? _____

AT THE LABORATORY:

127. In a typical week, about how many hours do you use a computer for your research? _____

128. How many computers do you have at the lab? _____

129. How many people use a computer at the lab? _____

130. How many of these lab computers are Internet connected? _____

**CELLPHONE USE**

131. Do you have a cell phone?  1=Yes  0=No

**If yes,**

132. In a day, about how many hours do you use your cell phone for research? _____

133. … about how many calls related to research do you receive? _____

134. … about how many calls related to research do you make? _____

135. How many years have you had a cell phone? _____

# WITH YOUR CELLPHONE, HAVE YOU EVER DISCUSSED RESEARCH WITH….

136.…graduate students?  1=Yes  0=No

137.…a professor*?  1=Yes  0=No

138.…my major professor/mentor?  1=Yes  0=No

139.…a colleague in another location in Japan?  1=Yes  0=No

140.…a colleague in Asia but outside Japan?  1=Yes  0=No

141.…a colleague in the developed countries (e.g. U.S., U.K)?  1=Yes  0=No

142.…a colleague in the developing countries (e.g. Indonesia, Philippines)?  1=Yes  0=No

143.…funding agencies?  1=Yes  0=No

**EMAIL USE**

FOR THESE NEXT QUESTIONS, THINK ABOUT YOUR **TYPICAL WEEK**:

144. How many hours do you spend receiving and sending email? _________

145. How many email messages do you send?

1 = Less than one a week

2 = Less than two per day

3 = Three to 10 per day

4 = More than ten daily

146. How many of these messages you send are related to your research?

1 = Less than one a week

2 = Less than two per day

3 = Three to 10 per day

4 = More than ten daily

147. How many email messages (EXCLUDE JUNK MAIL) do you receive?

1 = Less than one a week

2 = Less than two per day

3 = Three to 10 per day

4 = More than ten daily

148. How many of these messages you receive are related to your research?

1 = Less than one a week

2 = Less than two per day

3 = Three to 10 per day

4 = More than ten daily

# HAVE YOU EVER DONE THE FOLLOWING ON EMAIL?

149. Discussed research with other graduate students  1=Yes  0=No

150. Discussed research with a professor*  1=Yes  0=No

151. Discussed research with my major professor/mentor  1=Yes  0=No

152. Discussed research with a colleague in another location in Japan  1=Yes  0=No

153. Discussed research with a colleague in Asia but outside the Japan  1=Yes  0=No

154. Discussed research with a colleague in the developed countries (e.g. U.S., U.K)  1=Yes  0=No

155. Discussed research with a colleague in the developing countries (e.g. Indonesia, Philippines)  1=Yes  0=No

156. Started a professional relationship with someone you met through email  1=Yes  0=No

157. Reviewed a manuscript for journals  1=Yes  0=No

158. Discussed proposals with funding agencies  1=Yes  0=No

159. Submitted manuscripts for journal  1=Yes  0=No

**WEB USE**

160. In a typical week, how many hours do you spend using the web? __________

161. In a typical week, how many hours are spent on matters related to your research? __________

WHICH OF THE FOLLOWING HAVE YOU DONE ON-LINE?

162.…purchased a product or service for your research  1=Yes  0=No

163.…created a research project homepage  1=Yes  0=No

164.…conducted an information search  1=Yes  0=No

165.…used scientific research data  1=Yes  0=No

166.…collaborated on a scientific project  1=Yes  0=No

167.…conducted Internet-based conference or meeting for your research  1=Yes  0=No

168.…consulted another researcher through on-line telephony (e.g. Skype)  1=Yes  0=No

169.…found and examined reference materials (e.g. dictionary, encyclopedia)  1=Yes  0=No

170.…accessed research reports or scientific papers  1=Yes  0=No

171.…downloaded research software  1=Yes  0=No

172.…used an on-line data analysis software  1=Yes  0=No

**MENTORING PROBLEMS AND WHAT MENTORS DO**

The following is a list of problems that graduate students say they experience when working with their advisers. Based on your experience, is this a problem for you? Please evaluate each of these problem areas using the following scale: 1 = not a problem, 5 = major problem.

| Problems/Concerns | | Rating | | | | | | | | | | | | | | |
| --- | --- | --- | --- | --- | --- | --- | --- | --- | --- | --- | --- | --- | --- | --- | --- | --- |
|  | | NP |  | | |  | | |  | | | MP | | |  | |
| 173. Adviser rarely present in the lab | | 1 | | | 2 | | | 3 | | | 4 | | | 5 | | |
| 174. Adviser does not provide feedback on research work | | 1 | | | 2 | | | 3 | | | 4 | | | 5 | | |
| 175. Adviser uses computer and Internet for non-research purposes | | 1 | | | 2 | | | 3 | | | 4 | | | 5 | | |
| 176. Adviser not maintaining organized laboratory notes and research data | | 1 | | | 2 | | | 3 | | | 4 | | | 5 | | |
| 177. Adviser disrespectful to students | | 1 | | | 2 | | | 3 | | | 4 | | | 5 | | |
| 178. Adviser disrespectful to other professors | | 1 | | | 2 | | | 3 | | | 4 | | | 5 | | |
| 179. Adviser does not give advice on what to do | | 1 | | | 2 | | | 3 | | | 4 | | | 5 | | |
| 180. Adviser not in good relationship with other professors | | 1 | | | 2 | | | 3 | | | 4 | | | 5 | | |
| 181. Adviser not in lab meetings | | 1 | | | 2 | | | 3 | | | 4 | | | 5 | | |
| 182. Adviser does not listen to students opinions | | 1 | | | 2 | | | 3 | | | 4 | | | 5 | | |
| 183. Adviser not sensitive to students’ needs | | 1 | | | 2 | | | 3 | | | 4 | | | 5 | | |
| 184. Adviser not interested in students’ well-being | | 1 | | | 2 | | | 3 | | | 4 | | | 5 | | |
| 185. Adviser not available for consultations | | 1 | | | 2 | | | 3 | | | 4 | | | 5 | | |
| 186. Adviser difficult to communicate with orally and written | | 1 | | | 2 | | | 3 | | | 4 | | | 5 | | |
| 187. Adviser frequently on trips | | 1 | | | 2 | | | 3 | | | 4 | | | 5 | | |
|  | Rate your adviser in terms of the frequency of the following practices using the following scale: 1 = never, 2 = rarely, 3 = often, 4 = very often. Since 2008, how frequently your adviser done the following: | | | | | | | | | | | | | | |  |
|  |  | | | N | | |  | | |  | | | VO | | |  |
|  | 188. Adviser discusses career aspirations and plans with students | | | 1 | | | 2 | | | 3 | | | 4 | | |  |
|  | 189. Adviser monitors students’ work progress | | | 1 | | | 2 | | | 3 | | | 4 | | |  |
|  | 190. Adviser discusses students’ concerns and problems about research | | | 1 | | | 2 | | | 3 | | | 4 | | |  |
|  | 191. Adviser discusses personal and/or family problems with students | | | 1 | | | 2 | | | 3 | | | 4 | | |  |
|  | 192. Adviser co-authors research paper or book chapter with students | | | 1 | | | 2 | | | 3 | | | 4 | | |  |
|  | 193. Adviser co-directs research project with students | | | 1 | | | 2 | | | 3 | | | 4 | | |  |
|  | 194. Adviser analyzes data and performs calculations side-by-side with students | | | 1 | | | 2 | | | 3 | | | 4 | | |  |
|  |  | | | N | | |  | | |  | | | VO | | |  |
|  | 195. Adviser run experiments side-by-side with students | | | 1 | | | 2 | | | 3 | | | 4 | | |  |
|  | 196. Adviser reviews students for general and comprehensive exams | | | 1 | | | 2 | | | 3 | | | 4 | | |  |
|  | 197. Adviser helps students draft job application letters | | | 1 | | | 2 | | | 3 | | | 4 | | |  |
|  | 198. Adviser helps students draft their curriculum vitae | | | 1 | | | 2 | | | 3 | | | 4 | | |  |
|  | 199. Adviser helps students prepare for a job talk or presentation | | | 1 | | | 2 | | | 3 | | | 4 | | |  |
|  | 200. Adviser helps students search for job positions and announcements | | | 1 | | | 2 | | | 3 | | | 4 | | |  |
|  | 201. Adviser socializes students to members of the professional community | | | 1 | | | 2 | | | 3 | | | 4 | | |  |
|  | 202. Adviser gives feedback on students’ research and performance | | | 1 | | | 2 | | | 3 | | | 4 | | |  |

**GRADUATE TRAINING PRACTICES**

| Please rate the following practices in terms of their frequency using the following scale:  1 = never, 2 = rarely, 3 = often, 4 = very often. Since 2008, how frequently have you done the following: | Rating  N VO | | | |
| --- | --- | --- | --- | --- |
| 1. Present research in departmental seminars | 1 | 2 | 3 | 4 |
| 1. Present research in conferences (national, regional, international) | 1 | 2 | 3 | 4 |
| 1. Participate in research competitions (national, regional, international) | 1 | 2 | 3 | 4 |
| 1. Attend trainings to enhance research skills and techniques | 1 | 2 | 3 | 4 |
| 1. Organize professional meetings and conferences | 1 | 2 | 3 | 4 |
| 1. Perform data analyses | 1 | 2 | 3 | 4 |
| 1. Write and submit grant proposals | 1 | 2 | 3 | 4 |
| 1. Take the lead in a research lab meeting | 1 | 2 | 3 | 4 |
| 1. Review and comment on manuscripts which you were/are reviewing | 1 | 2 | 3 | 4 |
| 1. Write papers for submission to scholarly journals | 1 | 2 | 3 | 4 |
| 1. Draft letters to the editor for submission of manuscripts | 1 | 2 | 3 | 4 |
| 1. Draft responses to reviewers for revised and resubmitted manuscripts | 1 | 2 | 3 | 4 |
| 1. Visit other laboratories to learn skills and techniques | 1 | 2 | 3 | 4 |
| 1. Draft operating manuals for lab instruments and equipment | 1 | 2 | 3 | 4 |
| 1. Have senior students help junior students in their research | 1 | 2 | 3 | 4 |
| 1. Review and comment on reports and papers produced by the laboratory | 1 | 2 | 3 | 4 |
| 1. Review and critique recently published leading research articles | 1 | 2 | 3 | 4 |

**INTERACTION WITH ADVISER**

The following is a list of bi-polar adjectives that describes advisers’ interactions with their graduate students. Please rate each pair of adjectives based on your own personal experiences as a graduate student.

| *220. Face-to-face* | -3 | -2 | -1 | 0 | 1 | 2 | 3 | *Technology-mediated* |
| --- | --- | --- | --- | --- | --- | --- | --- | --- |
| *221. Formal/Impersonal* | -3 | -2 | -1 | 0 | 1 | 2 | 3 | *Informal/Personal* |
| *222. Frequent* | -3 | -2 | -1 | 0 | 1 | 2 | 3 | *Seldom* |
| *223. One-way* | -3 | -2 | -1 | 0 | 1 | 2 | 3 | *Two-way* |
| *224. To look forward to* | -3 | -2 | -1 | 0 | 1 | 2 | 3 | *To avoid* |
| *225. Exciting* | -3 | -2 | -1 | 0 | 1 | 2 | 3 | *Boring* |
| *226. Quick talks* | -3 | -2 | -1 | 0 | 1 | 2 | 3 | *Long discussions* |
| *227. Structured/hierarchical* | -3 | -2 | -1 | 0 | 1 | 2 | 3 | *Unstructured/non-hierarchical* |
| *228. Supportive* | -3 | -2 | -1 | 0 | 1 | 2 | 3 | *Adversarial* |
| *229. Friendly* | -3 | -2 | -1 | 0 | 1 | 2 | 3 | *Unfriendly* |
| *230. Considerate* | -3 | -2 | -1 | 0 | 1 | 2 | 3 | *Inconsiderate* |
| *231. General/Broad* | -3 | -2 | -1 | 0 | 1 | 2 | 3 | *Specifics/Detailed* |
| *232. Planned* | -3 | -2 | -1 | 0 | 1 | 2 | 3 | *Unplanned* |

**WORKING IN THE RESEARCH LABORATORY**

The following is a list of bi-polar adjectives that describes work environment in a research laboratory. Please rate each pair of adjectives based on your own personal experiences in such a setting.

| *233. Many things to learn* | -3 | -2 | -1 | 0 | 1 | 2 | 3 | *Nothing to learn* |
| --- | --- | --- | --- | --- | --- | --- | --- | --- |
| *234. Many things to do* | -3 | -2 | -1 | 0 | 1 | 2 | 3 | *Nothing to do* |
| *235. Competitive* | -3 | -2 | -1 | 0 | 1 | 2 | 3 | *Cooperative* |
| *236. To look forward to* | -3 | -2 | -1 | 0 | 1 | 2 | 3 | *To avoid* |
| *237. Exciting* | -3 | -2 | -1 | 0 | 1 | 2 | 3 | *Boring* |
| *238. Structured/hierarchical* | -3 | -2 | -1 | 0 | 1 | 2 | 3 | *Unstructured/non-hierarchical* |
| *239. Busy* | -3 | -2 | -1 | 0 | 1 | 2 | 3 | *Relaxed* |
| *240. Supportive* | -3 | -2 | -1 | 0 | 1 | 2 | 3 | *Adversarial* |
| *241. Friendly* | -3 | -2 | -1 | 0 | 1 | 2 | 3 | *Unfriendly* |
| *242. Open communication* | -3 | -2 | -1 | 0 | 1 | 2 | 3 | *Close communication* |
| *243. Authoritarian* | -3 | -2 | -1 | 0 | 1 | 2 | 3 | *Democratic* |
| *244. Has privacy* | -3 | -2 | -1 | 0 | 1 | 2 | 3 | *Has no privacy* |
| *245. Low supervision* | -3 | -2 | -1 | 0 | 1 | 2 | 3 | *High supervision* |

**OTHER INFORMATION**

246. What is your nationality?

 (1) Japanese  (2) Singaporean  (3) Taiwanese  (4) Chinese

 (5) Korean  (6) others, please specify: ______________________

247. In a typical week, how often do you go to church for mass or religious services?

 (1) Never

 (2) Once a week

 (3) Two to three times a week

 (4) Four to five times a week

 (5) Everyday

**Thanks very much for your time.**

**INTERVIEWER’S OBSERVATION**

248. What was respondent’s initial attitude about being interviewed?

 (1) Very interested

 (2) Somewhat interested

 (3) Indifferent

 (4) Somewhat reluctant

 (5) Very reluctant

 (6) Hard to tell

249. Was anyone else present during the interview?

 (1) Yes  (2) No

250. How open and forthcoming do you think respondent was about his/her feelings?

 (1) Very open

 (2) Held back somewhat

 (3) Held back a great deal

251. Did you observe any signs of tension or stress in respondent’s behavior?

 (1) Yes  (2) No

252. Where was the interview held?

 (1) Private setting (respondent’s own room and not shared with others)

 (2) Semi-private setting (respondent’s own room but shared with others)

 (3) Public setting (library, cafeteria, lobby; laboratory)
